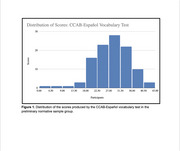# An Adaptive Vocabulary Test In Spanish

**DOI:** 10.1002/alz70857_107484

**Published:** 2025-12-26

**Authors:** Isabella Jaramillo, Kathleen Hall, Lourdes Anllo‐Vento, Kristin Geraci, Michael Blank, Miranda Miranda, Alejandra Ortiz‐Menchaca, Elloise Garcia, Enriqueta Canseco‐Gonzalez, Analia Arevalo, Peter Pebler, David L. Woods

**Affiliations:** ^1^ Neurobehavioral Systems, Inc, Berkeley, CA, USA; ^2^ Universidad de Granada, Granada, Granada, Spain; ^3^ Reed College, Portland, OR, USA; ^4^ University of São Paulo, São Paulo, São Paulo, Brazil

## Abstract

**Background:**

Vocabulary tests assess premorbid verbal intelligence, and vocabulary scores are usually more powerful predictors of cognitive test performance than education or other demographic characteristics. The California Cognitive Assessment Battery (CCAB) [1] includes an adaptive staircase vocabulary test in English. Here, we describe the development of an equivalent test in CCAB‐Español, the Spanish language CCAB. Preliminary data shows that scores on the CCAB‐Español vocabulary test are strong predictors of overall task performance on the Spanish battery.

**Method:**

CCAB‐Español is a carefully adapted version of the English CCAB, designed to ensure linguistic, dialectal, and cultural relevance of test items. The vocabulary test includes 300 possible target words across 48 difficulty levels. In 24 trials, participants see one stimulus word and four possible definitions, selecting the best match. All participants start at level 16, with difficulty adjusting up or down based on accuracy using a staircase with variable step sizes.

Creation of this test included careful contributions from a team of native Spanish‐speaking neuropsychologists. Levels were based on appropriate ranges of overall word frequency, and target words were selected for each level based on that range. Dialectal and regional frequencies were cross‐referenced using two databases with country‐specific data: the CORPES XXI 2021 corpus and the Corpus del Español. The test floor was also lowered to address potential differences in distribution, and ensure broad applicability across Spanish‐speaking communities.

**Results:**

Test instructions were presented with text‐to‐speech voices in a Mexican dialect. Participants (*n* = 108; age 18‐79; 62% women, 22% Central American, 3% Caribbean, 5% Spanish, 31% Mexican, 28% South American, 10% other) completed the CCAB‐Español vocabulary task (M=4.3 minutes) during the course of normative data collection.

Preliminary data indicates the CCAB‐Español vocabulary test is a strong predictor of overall performance on the Spanish battery with similar influences of vocabulary scores as in the English version of the CCAB.

**Conclusion:**

Vocabulary is a key measure of premorbid intelligence and is a stronger predictor of performance than education. CCAB‐Español's vocabulary test design incorporates linguistic and dialectal differences to ensure its broad applicability across Spanish‐speaking regions.